# Dental caries and bacterial load in saliva and dental biofilm of type 1 diabetics on continuous subcutaneous insulin infusion

**DOI:** 10.1590/1678-7757-2017-0500

**Published:** 2018-05-07

**Authors:** Ana COELHO, Anabela PAULA, Marta MOTA, Mafalda LARANJO, Margarida ABRANTES, Francisco CARRILHO, Manuel FERREIRA, Mário SILVA, Filomena BOTELHO, Eunice CARRILHO

**Affiliations:** 1 Universidade de Coimbra, Faculdade de Medicina iCBR, Coimbra, Portugal.; 2 Universidade de Coimbra, Faculdade de Medicina, Departamento de Microbiologia, Coimbra, Portugal.; 3 Centro Hospitalar e Universitário de Coimbra, Departamento de Endocrinologia, Diabetes e Metabolismo, Coimbra, Portugal.; 4 Universidade do Porto, Faculdade de Medicina Dentária, Porto, Portugal.

**Keywords:** Diabetes, Hyperglycemia, Dental caries, Saliva, Biofilm, Bacteria

## Abstract

**Objectives:**

Since most of the studies evaluates diabetics on multiple daily injections therapy and continuous subcutaneous insulin infusion may help gain better metabolic control and prevent complications, the objective of this study was to evaluate the prevalence of dental caries, the unstimulated salivary flow rate and the total bacteria load, *Streptococcus* spp. levels and *Lactobacillus* spp. levels in saliva and supragingival dental biofilm of type 1 diabetics on insulin pump.

**Material and Methods:**

Sixty patients with type 1 diabetes on insulin pump and 60 nondiabetic individuals were included. The dental caries evaluation was performed using ICDAS and the oral hygiene was assessed according to Greene and Vermillion Simplified Oral Hygiene Index. Unstimulated saliva and supragingival dental biofilm were collected. Total bacteria, *Streptococcus* spp. and *Lactobacillus* spp. was quantified by qPCR.

**Results:**

Patients with type 1 diabetes had a higher prevalence of dental caries and filled and missing teeth when compared with the control group. These patients were associated with more risk factors for the development of dental caries, namely a lower unstimulated salivary flow rate and a higher bacterial load in saliva and dental biofilm.

**Conclusion:**

Some risk factors related to dental caries were associated with type 1 diabetics. An early diagnosis combined with the evaluation of the risk profile of the diabetic patient is imperative, allowing the dental caries to be analyzed through a perspective of prevention and the patient to be integrated into an individualized oral health program.

## Introduction

Diabetes mellitus classification establishes the existence of four distinct types: type 1 diabetes, type 2 diabetes, gestational diabetes and other specific types of diabetes. Type 1 diabetes includes the cases in which the destruction of β-pancreatic cells occurs by autoimmune processes and those in which the etiology and pathogenesis of the pancreatic destruction is idiopathic^2^.

The long-term complications of type 1 diabetes include several micro and macrovascular changes combined with different risk factors, such as hyperglycemia, high glycemic variability, hypertension and duration of the disease. These vascular changes, especially the microvascular ones, have repercussions in several organs^34^.

Intensive insulin therapy aims to restore insulin levels through the administration of exogenous insulin and may be performed through multiple daily injections or Continuous Subcutaneous Insulin Infusion (CSII)^19^.

A CSII system consists of a small portable electromechanical device (the insulin pump), capable of continuously administrate insulin through a small catheter, in order to mimic the physiological secretion^22^.

The advantages of CSII over multiple daily injection therapy in patients with type 1 diabetes include a reduction in hypoglycemic episodes, a better control of early-morning increase in blood glucose (Dawn phenomenon) and a reduction of glycated hemoglobin (HbA_1_c) levels, with consequent improvement of patients’ quality of life^24^.

Among the oral complications associated with type 1 diabetic patients are changes in the salivary flow rate and composition, dental caries, periodontal disease and oral candidiasis. There is also a higher prevalence of burning mouth syndrome, aspergillosis, lichen planus, geographic tongue, postsurgical infections, halitosis and benign parotid enlargement, as well as taste disorders^16^. However, most of the complications are not adequately represented in the literature. Regarding dental caries, the results are controversial. Some authors reported a higher prevalence of dental caries in diabetic patients^1^ ^,^ ^21^ ^,^ ^23^, but others reported a similar^3^ ^,^ ^25^ or even a lower prevalence^13^ ^,^ ^26^.

Since most of the studies evaluates diabetics on multiple daily injections therapy and CSII may help obtain better metabolic control and prevent complications, the objective of this study was to evaluate the prevalence of dental caries, the unstimulated salivary flow rate and the total bacteria load, *Streptococcus* spp. levels and *Lactobacillus* spp. levels in saliva and supragingival dental biofilm of type 1 diabetics on insulin pump.

## Material and methods

This study was approved by the Ethics Committee of the Faculty of Medicine of the University of Coimbra. Informed consent was obtained from all individual participants included in the study.

Sixty patients with type 1 diabetes on CSII (test group), consulted at the Department of Endocrinology, Diabetes and Metabolism of the Coimbra Hospital and University Center, and 60 nondiabetic subjects (control group) were included in the study.

Patients with systemic or oral diseases related to salivary flow rate changes, with a body mass index above 25 kg/m^2^, undergoing orthodontic treatment, pregnant women, smokers and those who had used antibiotics or oral antimicrobials in the last 3 months were excluded. The inclusion criteria for the test group also comprised an established diagnosis of type 1 diabetes and treatment with CSII for at least 2 years. The subjects of the control group were friends or relatives of the diabetic patients or of other patients consulted at the Faculty of Medicine of the University of Coimbra. Patients diagnosed with any type of diabetes were excluded from the control group. Each diabetic patient was matched with a control of the same sex and age.

Diabetics’ metabolic control was assessed by HbA_1_c measurement, which was evaluated by the Department of Endocrinology, Diabetes and Metabolism of the Coimbra Hospital and University Center.

The assessment of dental caries was performed using ICDAS^31^. ICDAS codes 1 and 2 were grouped, since both represent incipient lesions. Cavitated lesions with visible dentin (ICDAS codes 5 and 6) were also grouped. The sum of the number of teeth/surfaces with ICDAS codes 1 to 6 and the sum of the number of teeth/surfaces with ICDAS codes 3 to 6 were also evaluated.

Oral hygiene was evaluated using the Simplified Oral Hygiene Index from Greene e Vermillion (OHI-S)^12^ (1939).

Unstimulated whole saliva and supragingival dental biofilm were collected from both groups. Patients were previously advised not to eat, drink, brush their teeth or smoke for at least 2 hours prior to sampling. The appointments were conducted during the morning.

The collection of saliva was performed according to the literature^14^. The total volume of saliva expelled was recorded in order to calculate the unstimulated salivary flow rate of each patient.

The dental biofilm was collected from the vestibular surface of the right upper first molar with the aid of a periodontal curette (Hu-Friedy; Chicago, Illinois, USA). In the case of the nonexistence of the first right upper molar, the sample was collected from the contralateral tooth and, in the case of its nonexistence, from the upper second molars.

DNA was extracted from saliva and biofilm samples by adding bacteria lysis buffer (Roche^®^ Molecular Systems; Pleasanton, California, USA) plus proteinase K. The mixtures were incubated for 1 h at 65°C, followed by an in-house extraction protocol based on DNA precipitation and isolation with cold ethanol. Total bacterial load, *Streptococcus* spp. Load, and *Lactobacillus* spp. load were quantified by real-time PCR (qPCR), using primers and targeting conserved bacterial rDNA sequence in the LightCycler^®^ 2.0 instrument (Roche^®^ Molecular Systems; Pleasanton, California USA) by SYBR green detection. Samples were tested in triplicate.

Data analysis was performed using IBM^®^ SPSS^®^ v.22.0 (IBM Corporation; Amrnonk, New York, USA) and the significance level was set at 5%. Comparison of quantitative variables between the two groups was made using t-Student and Man-Whitney tests. Normality distribution of quantitative variables was assessed using Shapiro-Wilk test. For analysis of nominal variables, the chi-square test was used. The Pearson and Spearman correlation tests were used to determine the strength of the relationships between variables.

## Results

Twenty-four men and thirty-six women, with a mean age of 34.63±12.91 years, were included in each group.

Regarding metabolic control, 55% of the patients with diabetes were considered poorly controlled (HbA_1c_>7.5%).

Regarding oral hygiene habits, no differences were found between groups. Most patients reported brushing their teeth at least twice a day and not using dental floss (Table 1).

**Table 1:** Tooth brushing and dental flossing frequency

		Diabetics		Control		p
		%	n	%	n	
Tooth brushing	1x/day	20%	12	15%	9	0.632
	≥2x/day	80%	48	85%	51	
Dental flossing	Not using	63.3%	38	63.3%	38	1
	≥1x/week	33.3%	20	33.3%	20	
	≥1x/day	3.3%	2	3.3%	2	

Half of the patients with type 1 diabetes reported having hypoglycemic episodes at night, but only one reported performing tooth brushing after sugar intake.

The diabetic patients had a greater number of decayed teeth than individuals from the control group (Table 2), as well as a greater number of filled teeth (test group: 5.15±3.95, control group: 3.55±3.00, p=0.033). No significant statistical differences were found regarding missing teeth (test group: 3.73±4.79, control group: 2.90±4.34, p=0.352).

**Table 2:** Distribution of dental caries pattern as *per* ICDAS codes

	ICDAS code	Diabetics	Control	p*
		n=60	n=60	
Surfaces	1-2	4.15±3.25	2.13±1.44	0.000
	3	0.77±1.41	0.43±0.95	0.185
	4	0.80±1.41	0.93±1.46	0.761
	5-6	1.00±2.38	0.85±3.44	0.323
	1-6	6.71±5.99	4.35±5.31	0.007
	3-6	4.77±6.07	2.56±3.89	0.003
Teeth	1-6	4.83±3.73	3.08±2.19	0.008
	3-6	1.72±2.29	1.28±1.76	0.402

* T-student

Correlation between HbA_1_c and the number of decayed, missing and/or filled teeth/surfaces was not found (p>0.05). Likewise, no correlation was found between the time elapsed since the diagnosis of type 1 diabetes and the number of decayed, missing and/or filled teeth/surfaces (p>0.05).

Regarding OHI-S, significant statistical differences between groups were not found (p=0.501). In the group of patients with diabetes, it was 1.66±1.11, and in the control group, it was 1.41±0.75. No correlation was found between HbA_1_c and OHI-S (rs=0.130, p=0.322) or the time elapsed since the diagnosis of diabetes (rs=0.031, p=0.815).

The diabetic patients had a lower salivary flow rate than the individuals of the control group (p=0.004). The unstimulated salivary flow rate of the diabetic patients was 0.309±0.049 ml/min and that of the control group was 0.335±0.047 ml/min.

Correlation between the metabolic control of the patients with diabetes and the unstimulated salivary flow rate was not found (rs=-0.002, p=0.987). Moreover, no correlation was found between the time elapsed since the diagnosis of type 1 diabetes and the unstimulated salivary flow rate (rs=-0.154, p=0.239).

**Figure 1:**
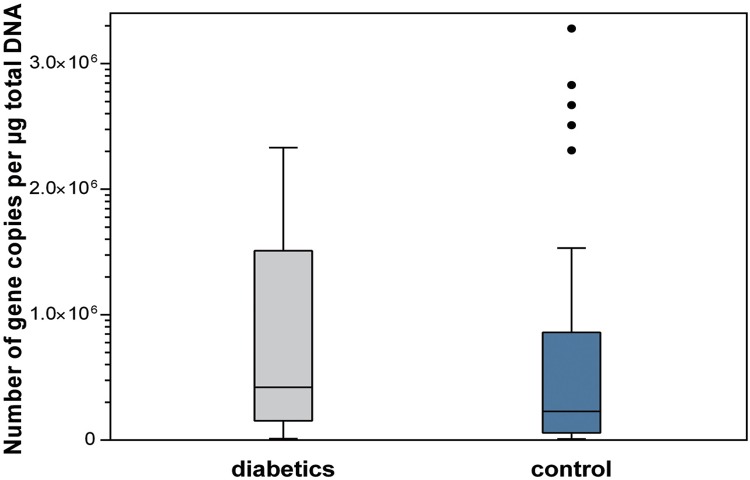
Salivary total bacterial load (p=0.036)

The diabetics had a higher salivary total bacterial load when compared with subjects of the control group. There were statistically significant differences between groups regarding salivary total bacteria load (p=0.036), *Streptococcus* spp. levels (p=0.050), and *Lactobacillus* spp. levels (p=0.050) (Figures 1 [Fig f02] to 3).

**Figure 2 f02:**
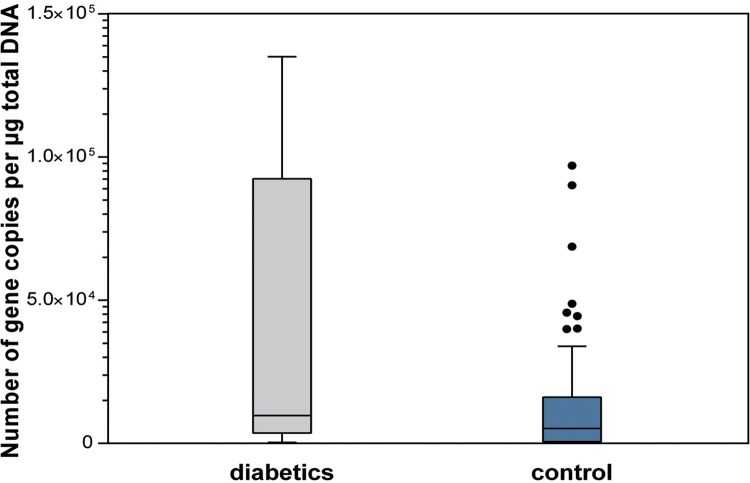
Salivary levels of *Streptococcus* spp (p=0.050)

**Figure 3:**
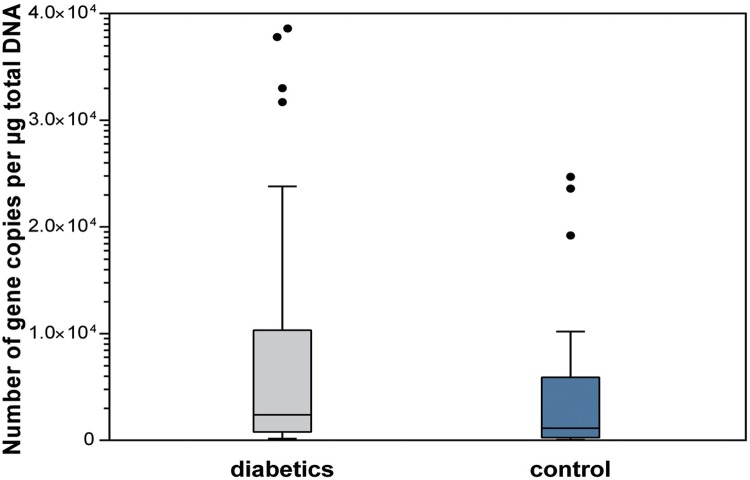
Salivary levels of *Lactobacillus* spp (p=0.050)

**Figure 4:**
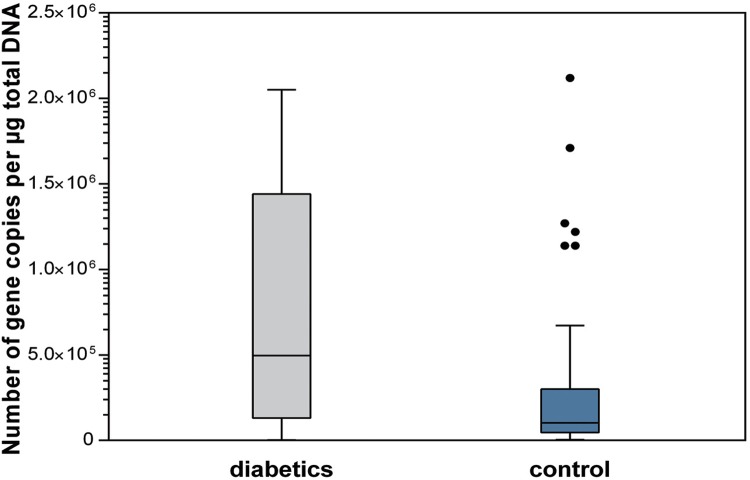
Total bacterial load in dental biofilm (p<0.001)

Regarding the dental biofilm, samples from patients with type 1 diabetes also had a higher total bacterial load when compared with those in the control group. Statistically significant differences were found between groups for total bacteria load (p<0.001), *Streptococcus* spp. levels (p=0.011), and *Lactobacillus* spp. levels (p=0.007) (Figures 4 [Fig f05] to 6).

**Figure 5 f05:**
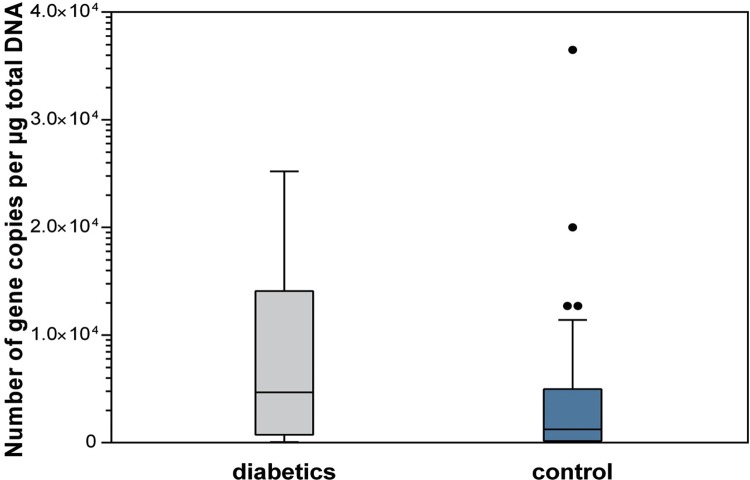
*Streptococcus* spp. levels in dental biofilm (p=0.011)

**Figure 6:**
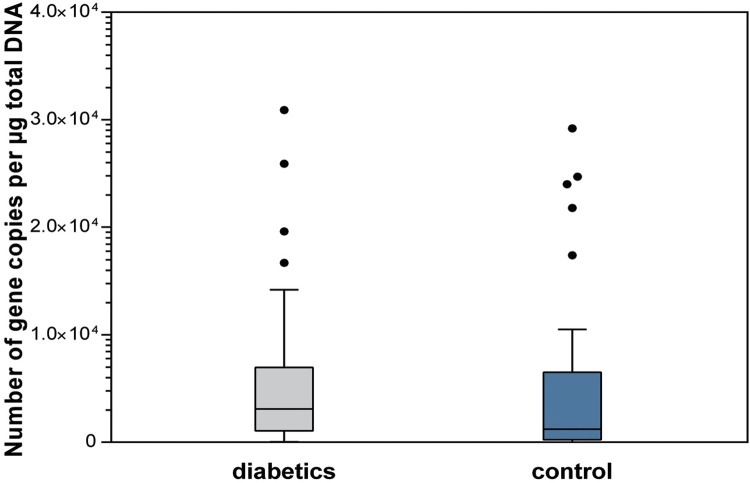
*Lactobacillus* spp. levels in dental biofilm (p=0.007)

No association was found between saliva and dental biofilm bacterial composition and HbA_1_c (p>0.05) or the time elapsed since the diagnosis of diabetes (p>0.05).

The patients with more than three decayed teeth (D_1-6_) had a higher total bacterial load in saliva (p=0.042), a higher load of *Streptococcus* spp. in saliva (p=0.050) and in dental biofilm (p=0.042), and a higher load of *Lactobacillus* spp. in saliva (p=0.029) and in dental biofilm (p=0.044).

## Discussion

Though 55% of the diabetic group were considered metabolic poorly controlled after HbA_1c_ evaluation, statistically significant differences between well and poorly controlled individuals were not found. Though HbA_1c_ is widely used, it represents an average, and there are important factors that do not significantly affect its value. In fact, there is clinical evidence that supports glycemic variability’s negative role in the development of diabetes complications, and HbA_1c_ evaluation does not allow inferring about it^5^.

The diabetic patients had a higher number of decayed teeth than individuals in the control group. These differences between groups were found to be statistically significant, in agreement with some studies found in the literature regarding patients on multiple insulin injections therapy^1^ ^,^ ^6^ ^,^ ^23^.

Teeth demineralization can be favored by a decrease in the salivary flow rate (which is often associated with the diabetic population) and promotes bacterial proliferation by reducing the rates of elimination of substrates and of dissolution of sugars, as well as less effective mechanisms of pH regulation and antimicrobial action^4^.

Several studies aimed to evaluate the salivary flow rate of patients with diabetes. Most authors associate these patients with a decrease in salivary flow rate when compared with nondiabetic individuals^7^ ^,^ ^18^ ^,^ ^20^, similarly to what happened in this study.

Busato, et al.^7^ (2012) reported a negative impact of the low salivary flow rate on the quality of life of a group of adolescents with type 1 diabetes. Contrary to the results of this study, some authors^6^ ^,^ ^8^ ^,^ ^20^ suggest a worsening of the situation with age and, consequently, with the duration of the disease. Some authors^6^ ^,^ ^8^ ^,^ ^20^ have also found a correlation between low salivary flow rate and poor metabolic control, but others^15^ ^,^ ^18^ ^,^ ^29^ did not find these correlations in their studies.

Among the several factors that are thought to be related to the decrease of the salivary flow rate in patients with diabetes, are the dehydration (related to hyperglycemia and consequent glycosuria), fatty acid infiltration in the salivary glands acinar cells, microvascular changes (with its consequent autonomic neuropathy), metabolic alterations and medication^18^ ^,^ ^32^.

Hypoglycemia resolution, through sugar ingestion, imposes the subsequent need for the patient’s education regarding oral hygiene, especially when it occurs during the night. Decrease in salivary flow rate during sleep delays the elimination of sugar from the oral cavity, decreases the rate of sugar dissolution and consequently favors its metabolization by the cariogenic bacteria^4^ ^,^ ^9^.

A high oral bacterial load can also be a risk factor, since the cariogenic bacteria are necessary for the development of dental caries^11^. Most studies aimed at assessing the microbiological composition of saliva in patients with type 1 diabetes only included children and adolescents in their test groups^1^ ^,^ ^25^ ^,^ ^27^ ^,^ ^28^, and there is controversy in the reported results.

Singh-Hüsgen et al.^25^ (2016) studied children and adolescents (aged 3 to 18 years) with and without type 1 diabetes and found higher concentrations of *S. mutans* and *Lactobacillus casei* in diabetics’ saliva, similarly to the results of this study. Likewise, Swanljung et al.^28^ (1992) reported a percentage of children with type 1 diabetes with a high salivary concentration of *S. mutans* (>10^5^ CFU/ml) higher than that found in a group of children without diabetes (control).

However, El-Tekeya et al.^10^ (2012) evaluated the non-stimulated saliva from a group of children with and without type 1 diabetes, and no differences between groups were found regarding salivary concentrations of *S. mutans* and *Lactobacillus* spp., as reported by other authors^1^ ^,^ ^27^.

The different inclusion criteria, the reduced size of some samples (as well as the consequent creation of groups with few elements after stratification by metabolic control) may, nevertheless, justify the lack of statistically significant results of some studies.

The importance of the study of the oral hygiene habits is clear since they represent an essential causal factor for the accumulation of dental biofilm^30^ ^,^ ^33^. Although a significant proportion of studies attribute higher plaque levels to patients with type 1 diabetes^17^ ^,^ ^23^ ^,^ ^25^, the evaluation of the oral hygiene habits is not performed in the majority of them.

Studies evaluating the bacterial composition of the dental biofilm of patients with type 1 diabetes were not found. In this study, the results of the microbiological analysis revealed that patients with type 1 diabetes have a higher total bacterial load in the dental biofilm. Moreover, a correlation between decayed, missing and/or filled teeth and the bacterial load in the dental biofilm was found, which demonstrates the importance of the dental biofilm as a risk factor for dental caries.

The correlation between the diabetics’ metabolic control and the susceptibility to dental caries reported by some authors requires a careful interpretation. The results regarding this association are controversial and the multifactorial etiology of the studied pathologies and their complications make it difficult to interpret the obtained data. On the other hand, behavioral factors may also play a critical role in this association and should therefore be considered. Oral health may be included in general health care, which may determine that a patient who attributes little importance to their oral health is also a patient who does not adequately adhere to the treatment of diabetes.

## Conclusions

An early diagnosis combined with the risk profile evaluation of the diabetic patient is imperative, allowing dental caries to be analyzed through a perspective of prevention. The clinician needs to know which risk factors for dental caries may be associated with patients with diabetes so that, after analysis, the patient can be integrated into an individualized oral health program, according to its risk profile.
